# Predictive factors of postoperative infection-related complications in adult patients with cerebral cavernous malformations

**DOI:** 10.1038/s41598-020-57681-9

**Published:** 2020-01-21

**Authors:** Chen-Yu Ding, Bao-Qiang Lian, Hong-Liang Ge, Qiu He, Ang Li, Xiao-Yong Chen, Jia-Heng Xu, Fu-Xin Lin, Yuan-Xiang Lin, De-Zhi Kang

**Affiliations:** Department of Neurosurgery, The First Affiliated Hospital of Fujian Medical University, Fuzhou, 350001 Fujian People’s Republic of China

**Keywords:** Predictive markers, Cerebrovascular disorders

## Abstract

Postoperative infection is an important factor contributing to poor prognosis after surgical treatment of cerebral cavernous malformations (CCM). However, the predictive factors of postoperative infection-related complications in adult patients with CCM have still not been well established. To identify possible predictive factors of postoperative infection after CCM surgery, we retrospectively evaluated the data of CCM patients who were enrolled into our prospective registry database. The relationship between preoperative characteristics of patients and postoperative infection-related complications was analyzed. A total of 167 CCM patients were included in this study. The average age was 39.69 ± 15.27 years old, and 21 of them had postoperative infection. For patients with postoperative infection, the Glasgow Coma Scale (GCS), Modified Rankin Scale (mRS), white blood cell (WBC) count, and neutrophil (NEU) count were all significantly higher than those of the group without infection. Our preliminary results showed that NEU count might have significant predictive value of intracranial infection, and GCS, mRS and CCM presenting with hemorrhage were all factors significantly related to postoperative pneumonia. Preoperative GCS, mRS and CCM presenting with hemorrhage might be used as predictive factors for postoperative pneumonia after CCM surgery, while preoperative NEU count can be used as an important predictive factor for postoperative intracranial infection after CCM surgery. Further large-scale studies are still needed to confirm this finding.

## Introduction

Cerebral cavernous malformation (CCM) is a benign cerebral vascular hamartoma commonly seem in neurosurgery. For CCM patient presenting with hemorrhage, having focal neurological malfunction or epilepsy, surgery is a routine treatment method^1–5^. In recent decades, advances in surgical techniques, postoperative care, and treatment strategies have improved clinical outcomes of CCM patients. However, there is still a certain percent of CCM patients having postoperative infection-related complications. These complications, including pneumonia and intracranial infection, are among the most serious and life-threatening complications after CCM surgery and are associated with poor short- and long-term outcomes^6,7^. For CCM patient with infection-related postoperative complications, a prolonged clinical course is needed. Therefore, early identification of these patients provides physicians the opportunity to discuss the clinical course with patient’s family, and for them to make informed decision on treatment options. This has clinical significance on improving the treatment results of CCM patients^8^. For CCM patients, there are several possible causes of postoperative infection-related complications and predicting such complications remains a challenge. Studies investigating the benefits of assessing clinical scores, [eg, Glasgow Coma Scale (GCS), Karnofsky Performance Scale (KPS) and Modified Rankin Scale (mRS)] and monitoring inflammation [eg, white blood cell (WBC) count and C-reactive protein level] to help in the prediction of postoperative infection-related complications have provided ambiguous results for many other types of cerebrovascular diseases, including aneurysmal subarachnoid hemorrhage and stroke^9–13^. Therefore, in this study, we sought to investigate the predictive factors of postoperative infection-related complications in patients with CCM.

## Materials and Methods

### Study population

Information of CCM patients admitted between January 2010 and December 2017 to the Department of Neurosurgery of the First Affiliated Hospital of Fujian Medical University was retrospectively reviewed. A total of 167 patients were included, the average age of patients was 39.69 ± 15.27 years, with 43.1% (72/167) being female.

The inclusion criteria were: (1) Age >18 years old; (2) diagnosed with CCM based on preoperative imaging, treated with surgery and confirmed with CCM based on postoperative pathological result. Exclusion criteria were: (1) Patient with incomplete disease information, such as missing any of the following: assessment of disease severity (including GCS, KPS and mRS) and WBC differential count (including the WBC count, neutrophil (NEU) count and lymphocyte (LYM) count) at admission, imaging and pathological information, and information on postoperative complications; (2) the patient had any type of surgery or acute or chronic infection within the past month; (3) prior onset of other neurological diseases such as intracranial tumors, stroke or severe head trauma; (4) patient had previous CCM surgery; (5) previous use of immunosuppressants; (6) other systemic diseases, such as autoimmune disease, uremia, cirrhosis, cancer, and chronic lung and heart diseases. The study conformed to the ethical guidelines of the Declaration of Helsinki. The local Ethics Committee of the First Affiliated Hospital of Fujian Medical University (Fujian, China) approved this study and waived the requirement for informed consent because of its retrospective design.

### Patient management

Clinical management and indications for surgery followed the guidelines from Angioma Alliance Scientific Advisory Board Clinical Experts Panel^14^. All patients had surgery to remove CCM, preventive antibiotic (cefazolin sodium) was injected at half an hour before surgery, and another dose was injected if the surgery extended beyond three hours. All patients received postoperative pump injection of sodium valproate to prevent epilepsy, and had an oral dose of it after its level reached stable in the blood. Budesonide and terbutaline were also administrated, via atomization therapy. The treatment of postoperative pneumonia followed the guidelines from the French Society of Anaesthesia and Intensive Care Medicine^15^.

### Clinical variables and detection of postoperative infection-related complications

Comprehensive data of each patient were collected, including information about medical history, history of current illness, admission clinical status, image files, treatments received, and all other data related to their hospitalization. Diagnosis of CCM was based on computerized tomography angiography (CTA) or MRI (magnetic resonance imaging). The location and size of the CCM was determined via MRI. As part of the routine patient care procedure, preoperative WBC differential count and clinical grades were obtained for each patient at admission. The variable “need for feeding tube” was defined as use of feeding tube before the occurrence of infection-related complications.

Diagnosis of postoperative pneumonia was based on the modified Centers for Disease Control and Prevention (CDC) criteria^16^. Postoperative pneumonia was diagnosed if patients developed lower respiratory tract infection within 30 days after surgical treatment. The patient could have possible postoperative pneumonia, which was defined by CDC as: meets the diagnosis criteria, but not confirmed by the admission or follow up chest X-ray (or X-ray examination was not performed), without other possible explanation of the symptoms or confirmed diagnosis of other diseases. The patient could also have confirmed postoperative pneumonia, which means the symptom met all CDC diagnosis criteria, including at least one chest X-ray confirmed diagnostic change. In the current study, both possible and confirmed pneumonia as determined by the CDC criteria were considered as pneumonia^17^.

Diagnosis of intracranial infection was based on the following criteria: (1) clinical manifestation of intracranial infection was observed; (2) patient with risk factors, such as human immunodeficiency virus/acquired immunodeficiency virus, hematopoietic stem cell transplant, lymphoid malignancies, neutropenia, hereditary immune defects, and patients with drainage or cerebrospinal fluid (CSF) leakage; (3) CSF parameters indicating for inflammation, such as WBC count >100 *10^6^/L and multinuclear leukocytes >70%, glucose levels <2.25 mmol/L and CSF glucose/serum glucose <0.66, and chloride <120 mmol/L and protein >0.45 g/L; 4) Positive CSF bacteria culture results, or presence of bacteria confirmed by polymerase chain reaction or other molecular biology techniques. Diagnosis of intracranial infection was made based on criteria 4 alone, or for patients without CSF bacteria culture result or with negative result but met all of criteria (1–3)^18,19^.

Based on the clinical data of this cohort of patients, the types of infection were categorized into pneumonia, intracranial infection, wound infection, bacteremia, or urinary tract infection. Primary site of infection was identified independently by two of the clinically trained researchers (BQL and CYD) based on the medical history, symptoms, physical examination, blood tests, X-rays, specimen cultures obtained from body sites other than blood, biopsy samples from surgical procedures, and autopsies. If for the same patient, the two researchers identified different site of infection, a third researcher (DZK) would make the final decision on this.

### Statistical analysis

In this study, statistical analysis was performed with SPSS 17.0 (SPSS Inc, Chicago, Illinois). Continuous variables were expressed as mean ± standard deviation and analyzed by 2-sample *t* test. Categorical variables were expressed as counts (percentage) and analyzed by Pearson χ^2^ test or Fisher exact test. Multivariate logistic regression model analysis was performed to assess predictors of postoperative infection-related complications, using the following steps: (1) For all available demographics and baseline variables that had univariate association of *P* < 0.05 with the occurrence of postoperative infection-related complications, univariate logistic regression analysis was performed; (2) All variables with *P* < 0.05 from univariate logistic regression analysis were included in multivariate analysis, and backward stepwise multivariate regression was performed to create the final model whereby the least nonsignificant variables were removed from the model one at a time, until all remaining variables had *P* < 0.05. Using the best threshold, which was derived from the receiver operating characteristic (ROC) curve analysis, the corresponding sensitivity and specificity of the predictive factors were also calculated. Area under the curve (AUC) model performance was evaluated using the *Z* test.

## Results

### Patient characteristics

167 patients were included in this study and categorized into infection-related complication group (n = 21) and no infection-related complication group (n = 146). Patient demographics, prior medical history, clinical characteristics, medical complications and inflammatory cell test results were compared between the two groups and results are shown in Table 1. Patients with postoperative infection-related complications had significantly higher WBC count, NEU count and clinical grades (GCS, KPS and mRS) at admission than those without (Table 1).

**Table 1:** Demographic features and clinical characteristics of patients with and without postoperative infection-related complications.

Characteristics	Total (n = 167)	Infection-related complications (n = 21)	No infection-related complications (n = 146)	*P* value	Intracranial infection (n = 6)	No intracranial infection (n = 161)	*P* value	Pneumonia (n = 12)	No pneumonia (n = 155)	*P* value
**Demographics**										
Age, yrs	39.69 ± 15.27	40.71 ± 14.41	39.55 ± 15.43	0.745	36.67 ± 15.70	39.81 ± 15.29	0.622	42.50 ± 14.92	39.48 ± 15.32	0.510
Gender, female	72 (43.1%)	7 (33.3%)	65 (44.5%)	0.333	4 (66.7%)	68 (42.2%)	0.404	2 (16.7%)	70 (45.2%)	0.055
**Admission clinical grade**										
GCS	15 (15–15)	15 (12–15)	15 (15–15)	0.002	15 (10.75–15)	15 (15–15)	0.462	12.5 (10.5–15)	15 (15–15)	0.001
KPS	80 (70–90)	80 (70–90)	80 (80–90)	0.072	80 (65–90)	80 (70–90)	0.468	70 (55–90)	80 (80–90)	0.108
mRS	1 (1–2)	2 (1–3.5)	1 (1–2)	0.003	2 (1–4)	1 (1–2)	0.087	2 (1–4)	1 (1–2)	0.027
**CCM characteristics**										
volume, cm^3^	5.08 ± 7.71	7.87 ± 9.96	4.67 ± 7.29	0.076	7.53 ± 7.73	4.99 ± 7.70	0.428	7.70 ± 7.94	4.87 ± 7.68	0.223
diameter, mm	1.98 ± 1.02	2.34 ± 1.09	1.93 ± 1.01	0.086	2.39 ± 1.16	1.96 ± 1.02	0.315	2.33 ± 1.02	1.95 ± 1.04	0.215
Deep locations	90 (53.9%)	14 (66.7%)	76 (52.1%)	0.209	5 (83.3%)	85 (52.8%)	0.219*	9 (75.0%)	81 (52.3%)	0.128
Brainstem (vs. other deep locations)	31 (18.6%)	6 (28.6%)	25 (17.1%)	0.208	0 (0.0%)	31 (19.3%)	0.235	5 (41.7%)	26 (16.8%)	0.033
Presenting with hemorrhage (vs. other symptoms)	51 (30.5%)	10 (47.6%)	41 (28.1%)	0.069	2 (33.3%)	49 (30.4%)	1.000*	8 (66.7%)	43 (27.7%)	0.008
Timing from hemorrhage to surgery, days	8 (5–16)	12 (4.5–16.25)	7 (5–15)	0.643	13 (10–16)	8 (5–15.5)	0.402	12 (6–16.5)	7 (5–15)	0.468
Need for feeding tube	39 (23.4%)	8 (38.1%)	31 (21.2%)	0.089	1 (16.7%)	38 (23.6%)	0.697	6 (50%)	33 (21.3%)	0.024
**Medical history**										
Hypertension	13 (7.8%)	2 (9.5%)	11 (7.5%)	0.669*	1 (16.7%)	12 (7.5%)	0.390*	1 (8.3%)	12 (7.7%)	1.000*
Diabetes mellitus	5 (3.0%)	1 (4.8%)	4 (2.7%)	0.494*	0 (0.0%)	5 (3.1%)	1.000*	1 (8.3%)	4 (2.6%)	0.314*
Smoking history	16 (9.6%)	3 (14.3%)	13 (8.9%)	0.429*	1 (16.7%)	15 (9.3%)	0.459*	1 (8.3%)	15 (9.7%)	1.000*
**Laboratory**										
WBC, ×10^9^/L	6.88 ± 2.23	7.81 ± 2.19	6.75 ± 2.21	0.041	8.80 ± 1.85	6.81 ± 2.22	0.032	7.71 ± 2.00	6.82 ± 2.24	0.186
NEU, ×10^9^/L	4.12 ± 1.95	5.01 ± 1.93	3.99 ± 1.92	0.025	5.77 ± 1.95	4.06 ± 1.93	0.034	4.83 ± 1.51	4.07 ± 1.97	0.191
LYM, ×10^9^/L	2.01 ± 0.95	1.96 ± 0.74	2.02 ± 0.97	0.772	2.18 ± 0.75	2.01 ± 0.95	0.671	2.00 ± 0.80	2.01 ± 0.96	0.972
RBC, ×10^12^/L	4.60 ± 0.50	4.54 ± 0.58	4.61 ± 0.50	0.566	4.87 ± 0.44	4.59 ± 0.51	0.184	4.43 ± 0.54	4.61 ± 0.50	0.254
HCT	0.41 ± 0.04	0.39 ± 0.04	0.41 ± 0.04	0.076	0.40 ± 0.02	0.41 ± 0.04	0.786	0.39 ± 0.05	0.41 ± 0.04	0.135
PLT × 10^9^/L	230.62 ± 70.03	232.38 ± 78.14	230.37 ± 69.07	0.903	269.50 ± 87.10	229.17 ± 69.22	0.167	229.08 ± 69.4	230.74 ± 70.30	0.937
HGB, g/L	136.72 ± 15.62	131.14 ± 15.25	137.52 ± 15.57	0.080	132.83 ± 14.98	136.86 ± 15.66	0.536	131.91 ± 16.73	137.09 ± 15.52	0.270

GCS: Glasgow Coma Scale, KPS: Karnofsky Performance Scale, mRS: Modified Rankin Scale, WBC: white blood cell, NEU: neutrophil, LYM: lymphocyte, RBC: red blood cell, HCT: hematocrit, PLT: blood platelet, HGB: hemoglobin.

The postoperative infection-related complications of CCM patients were shown in Table 2. In the postoperative infection-related complication group, the most commonly seen infection was pneumonia (57.1%, 12/21), all patients had positive result for their sputum microbial culture, including seven cases of streptococcus pneumoniae, two cases of acinetobacter baumannii, one case of staphylococcus aureus, one case of pseudomonas aeruginosa, and one case of candida albicans; the second commonly seen infection was intracranial infection (28.6%, 6/21), four out of six patients had positive lumbar puncture CSF microbial culture results, including one case of staphylococcus aureus, one case of staphylococcus epidermidis, one case of Klebsiella pneumoniae and one case of acinetobacter baumannii (Table 2).

**Table 2:** The postoperative infection-related complications of CCM patients.

Types of infection	No. of episodes of infection			Total (n = 167)
	<1 week	1–3 weeks	>3 weeks	
Pneumonia	5	3	4	12 (7.2%)*
Intracranial infection	3	3	0	6 (3.6%)*
Wound infection	0	2	0	2 (1.2%)
Bacteremia	1	1	0	2 (1.2%)
Urinary tract infection	1	0	0	1 (0.6%)
Subtotal	10 (43.5%)	9 (39.1%)	4 (17.4%)	

*There were two patients who had both pneumonia and intracranial infection.

### Predictive factors of postoperative infection-related complications

There were 21 patients (12.6%) who experienced infection-related complications after surgical treatment. Four preoperative variables (GCS, mRS, WBC count, and NEU count) that might be associated with postoperative infection-related complications were analyzed with univariate and multivariate regression analyses. The preliminary results showed that both mRS (odds ratio [OR] = 5.22, 95% confidence interval [CI] = 1.51–17.97, *P* = 0.009) and the NEU count (OR = 4.72, 95%CI = 1.76–12.68, *P* = 0.002) were significant predictors associated with postoperative infection-related complications (Table 3 and Supplementary Table 1).

**Table 3:** Univariate analyses of factors contributing to infection-related complications.

Predictors	Univariate analysis			
	Infectious complications (n = 21)	No infectious complications (n = 146)	OR (95% CI)	*P* value
GCS ≤ 13	9 (42.9%)	19 (13.0%)	5.01 (1.86–13.49)	0.001
mRS score ≥ 3	6 (28.6%)	9 (6.2%)	6.09 (1.90–19.47)	0.002
WBC ≥ 7.3 × 10^9^/L	13 (61.9%)	46 (31.5%)	3.53 (1.37–9.11)	0.009
NEU ≥ 4.7 × 10^9^/L	13 (61.9%)	35 (24.0%)	5.15 (1.98–13.45)	0.001

The cut-off points of predictors were calculated on the basis of ROC curve analysis. Backward stepwise regression methods were performed to create the final model whereby the least nonsignificant variable was removed from the model one at a time, until all remaining variables had *P* < 0.05.

**Figure 1:**
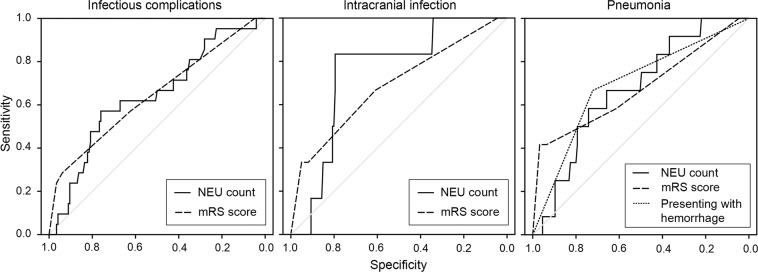
ROC curve analyses comparing the postoperative infectious complications predictive factors, the intracranial infection predictive factors and the pneumonia predictive factors. ROC curves were constructed on the basis of the sensitivity and specificity of the predictive factors. Comparisons of the AUC performances using *Z* test revealed that the power of NEU count was comparable with the mRS score in predicating the postoperative infectious complications (Z = 0.042, *P* = 0.966), the intracranial infection (Z = 0.434, *P* = 0.664), and the pneumonia (Z = 0.017, *P* = 0.987); and the power of presenting with hemorrhage was comparable with that of the NEU count (Z = 0.194, *P* = 0.846) and mRS score (Z = 0.151, *P* = 0.880) in predicating the postoperative pneumonia.

ROC curve analysis revealed that the NEU count for assessing the predictive performance of infection-related complications is represented as AUC = 0.642 (95%CI = 0.514–0.770), and the sensitivity and specificity were derived as 57.1% and 76.0%, respectively, based on the best threshold of 4.7 × 10^9^/L for NEU count (Fig. 1). The AUC for mRS was 0.646 (95%CI = 0.510–0.782). Comparison of the AUC performance using *Z* test method revealed that the predictive power of NEU count was comparable with that of mRS score (*Z* = 0.042, *P* = 0.966) (Fig. 1).

### Predictive factor of postoperative pneumonia

There were 12 patients (7.2%) who experienced pneumonia after surgical treatment. Five variables (GCS, mRS, brainstem CCM, CCM presenting with hemorrhage, and need for feeding tube) that might be associated with postoperative pneumonia were analyzed with univariate and multivariate regression analyses. The preliminary results showed that GCS (OR = 5.26, 95%CI = 1.33–20.79, *P* = 0.018), mRS (OR = 15.19, 95%CI = 3.08–74.86, *P* = 0.001) and CCM presenting with hemorrhage (OR = 4.25, 95%CI = 1.01–17.89, *P* = 0.049) were all significant predictors associated with postoperative pneumonia (Table 4 and Supplementary Table 2).

**Table 4:** Univariate and multivariate analyses of postoperative pneumonia-related factors*.

Predictors	Univariate analysis			
	Pneumonia (n = 12)	No pneumonia (n = 155)	OR (95% CI)	*P* value
GCS ≤ 13	7 (58.3%)	21 (13.5%)	8.93 (2.60–30.76)	0.001
mRS score ≥ 4	5 (41.7%)	5 (3.2%)	21.43 (5.01–91.62)	<0.001
Brainstem CCM	5 (41.7%)	26 (16.8%)	3.54 (1.04–12.04)	0.043
Presenting with hemorrhage	8 (66.7%)	43 (27.7%)	5.21 (1.49–18.19)	0.010
Need for feeding tube	6 (50.0%)	33 (21.3%)	3.70 (1.12–12.22)	0.032

The cut-off points of predictors were calculated on the basis of ROC curve analysis. Backward stepwise regression methods were performed to create the final model whereby the least nonsignificant variable was removed from the model one at a time, until all remaining variables had *P* < 0.05.

ROC curve analysis revealed that the NEU count for assessing the predictive performance of infection-related complications was represented as AUC = 0.674 (95%CI = 0.535–0.812), and the sensitivity and specificity were derived as 58.3% and 74.2%, respectively, based on the best threshold of 4.7 × 10^9^/L for NEU count (Fig. 1). The AUC for mRS and CCM presenting with hemorrhage were 0.676 (95%CI = 0.490–0.862) and 0.695 (95%CI = 0.535–0.855), respectively. The sensitivity and specificity were derived as 41.7% and 96.8% for mRS based on the best threshold of 3.5, and 66.7% and 72.3% for CCM presenting with hemorrhage, respectively.

Comparison of the AUC performances using *Z* test method revealed that the predictive power of NEU count was comparable with that of mRS (Z = 0.017, *P* = 0.987) and CCM presenting with hemorrhage (Z = 0.194, *P* = 0.846) (Fig. 1).

### Predictive factors of postoperative intracranial infection

There were six patients (3.6%) who experienced intracranial infection after surgical treatment.

Two variables (WBC count and NEU count) that might be associated with postoperative intracranial infection were analyzed with univariate and multivariate regression analyses. The preliminary results showed that NEU count ≥5.2 × 10^9^/L remained a significant predictor associated with the occurrence of postoperative intracranial infection (OR = 19.394, 95%CI = 2.190–171.713, *P* = 0.008) after adjusting for possible confounding factors (Table 5 and Supplementary Table 3).

**Table 5:** Univariate analyses of postoperative intracranial infection-related factors*.

Predictors	Univariate analysis			
	Intracranial infection (n = 6)	No intracranial infection (n = 161)	OR (95% CI)	*P* value
WBC ≥ 8.0 × 10^9^/L	5 (83.3%)	44 (27.3%)	13.30 (1.15–117.01)	0.020
NEU ≥ 5.2 × 10^9^/L	5 (83.3%)	33 (20.5%)	19.39 (2.19–171.71)	0.008

*Analyzed factors included all variable in Table 1 that had *P* < 0.05. The cut-off points of predictors were calculated on the basis of ROC curve analysis.

ROC curve analysis revealed that the NEU count for assessing the predictive performance of infection-related complications is represented as AUC = 0.751 (95%CI = 0.594–0.908), and the sensitivity and specificity were derived as 83.3% and 79.5%, respectively, based on the best threshold of 5.2 × 10^9^/L for NEU count (Fig. 1). The AUC for mRS was 0.688 (95%CI = 0.452–0.925). Comparison of the AUC performances using *Z* test method revealed that the predictive power of NEU count was comparable with that of mRS (Z = 0.434, *P* = 0.664) (Fig. 1).

## Discussion

Postoperative infection-related complications, either occurred during hospitalization or after discharge are common and severe complications of the patients with cerebrovascular diseases such as CCM, and could lead to worse outcomes^2^. In this study, it was found that for the group of patients with postoperative infection-related complications, their admission GCS, mRS, preoperative WBC and NEU counts were all significantly higher than those of the no infection group. Severity of CCM and NEU count might be useful predictors of postoperative infection-related complications of CCM patient, and might outperform many classic predictors, including age, gender, WBC count and smoking. Our preliminary results revealed that NEU count is an independent predictor of postoperative intracranial infection of CCM patients; GCS, mRS and CCM presenting with hemorrhage are independent predictors of postoperative pneumonia, after adjusting for confounders.

Previous research has shown that for diseases such as stroke, both the severity of the disease and blood inflammatory parameters are related to postoperative infection-related complications. Our results indicate it might also be true for CCM patients, as multivariate analysis revealed that degree of severity (mRS) and blood inflammatory factor (NEU count) were both independent predictors. For patients with intracranial infection, NEU count, not the severity of CCM, was the most significant factor; on the other hand, for pneumonia, severity of CCM (GCS, mRS, and CCM presenting with hemorrhage) was more significant. A possible explanation is that for patients with less severe CCM, even with high levels of blood inflammatory parameters, they might have a good recovery and become mobile early, leading to low pneumonia rate. Therefore, severity of CCM was a significant factor related to postoperative pneumonia; for patients with intracranial infection, there may be several potential explanations for the possible connection between the NEU count and infection. First, NEU count is a simple marker of subclinical infection. Although patients with any type of infectious events detected before admission were excluded, an elevated NEU count may indicate an underlying inflammatory process. Second, after CCM hemorrhage, immunologic changes happen as the early systemic inflammatory response, which is altered through the sympathetic pathway and the hypothalamus-pituitary-adrenal axis^20–22^. As a result, neutrophil demargination occurs^17,23^. Morever, for CCM patients presenting with hemorrhage, steroids may be used with edema and therefore increase the WBC. In the current study, patients with previous use of immunosuppressants, thus steroids were excluded. However, a published study^24^ has indicated preoperative steroid use is one of the predictors of any neurosurgery infection.

One of the critical factors impacting the results and prognosis of patients having neurological surgeries is hospital acquired infection^11,25^. Early prognosis of aSAH patients with postoperative pneumonia, whom are expected to have a prolonged clinical course will enable physicians to discuss with family members the expected clinical course of disease, for them to make informed decisions on treatment options^8^. Therefore, it is especially important to identify the high-risk CCM patients with postoperative infection-related complications. In the current study, ROC curve analysis revealed that the performance of NEU count as a predictor of infection-related complications was represented as AUC = 0.751 (95%CI = 0.594–0.908), and the sensitivity and specificity were derived as 83.3% and 79.5%, respectively, based on the best threshold of 5.2 × 10^9^/L for NEU count. For patients with elevated NEU count, preoperative education and strengthened respiratory tract management is suggested. Such interventions include smoking cessation, deep breathing exercise to improve respiratory function, mouth cleaning, strengthen nutrition by adjusting diet structure, and atomization to treat bronchial spasm. However, further research is still needed to validate this approach.

There were several limitations of this study, including its observational design. As a single center observational study, the inherent limitations of data analyses exist in this study. Although a relatively large number of patients were assessed in this study, there were only a relatively small number of patients with infection, which limits statistical analysis; the generalization of the findings to clinics should be progressed with caution. Further large-scale studies are still needed to confirm this finding.

## Summary

In conclusion, the severity of CCM and blood test parameters, which are easily obtained during the routine admission evaluation, may help to identify CCM patients with high risk of having postoperative infection-related complications. The preoperative GCS, mRS and CCM presenting with hemorrhage could be used as predictors of postoperative pneumonia after CCM surgery, while preoperative NEU count can be used as an important predictor of intracranial infection after CCM surgery.

## Supplementary information

Supplementary Tables.
