# Supplementary material for: Predictive factors of postoperative infection-related complications in adult patients with cerebral cavernous malformations
Source: Sci Rep. 2020 Jan 21;10:863. doi: 10.1038/s41598-020-57681-9 (PMC6972745; doi:10.1038/s41598-020-57681-9)
Supplement: Supplementary file 1 — Supplementary Tables. [file 41598_2020_57681_MOESM1_ESM.doc]

**Predictive factors of postoperative infection-related complications in adult patients with cerebral cavernous malformations**

Chen-Yu Ding^1*^, Bao-Qiang Lian^1*^, Hong-Liang Ge^1*^, Qiu He^1^, Ang Li^1^, Xiao-Yong Chen^1^, Jia-Heng Xu^1^, Fu-Xin Lin^1^, Yuan-Xiang Lin^1^, De-Zhi Kang^1^

^1^Department of Neurosurgery, The First Affiliated Hospital of Fujian Medical University, Fuzhou, 350001, Fujian, People's Republic of China.

* These authors should be considered co-first authors

**Corresponding author:** De-Zhi Kang, MD, PhD, Department of Neurosurgery, The First Affiliated Hospital of Fujian Medical University, Fuzhou, China. E-mail: kdz99999@sina.com

**Running Head:** Predictors of infection in CCM

**Number of supplementary tables:** 3

| Supplementary Table 1. Multivariate analyses of factors contributing to infection-related complications* | | |
| --- | --- | --- |
| Components of models | Multivariate analysis† | |
|  | OR (95% CI) | *P* value |
| Model I |  |  |
| GCS≤13 | 2.83(0.94-8.49) | 0.064 |
| mRS score ≥ 3 | 4.08(1.11-15.00) | 0.034 |
| WBC ≥ 7.3×10^9^/L | 0.81(0.17-3.88) | 0.796 |
| NEU≥ 4.7×10^9^/L | 4.60(0.98-21.60) | 0.053 |
| Final Model |  |  |
| mRS score ≥ 3 | 5.22 (1.51-17.97) | 0.009 |
| NEU≥ 4.7×10^9^/L | 4.72 (1.76-12.68) | 0.002 |
| *All variables having *P*<0.05 from univariate analysis were included in multivariate analysis. The cut-off points of predictors were calculated on the basis of ROC curve analysis. †Backward stepwise regression methods were performed to create the final model whereby the least nonsignificant variable was removed from the model one at a time, until all remaining variables had *P*<0.05. | | |

| Supplementary Table 2. Multivariate analyses of postoperative pneumonia-related factors* | | |
| --- | --- | --- |
| Components of models | Multivariate analysis† | |
|  | OR (95% CI) | *P* value |
| Model I |  |  |
| GCS ≤ 13 | 5.04(1.17-21.67) | 0.030 |
| mRS score ≥ 4 | 14.60(2.77-76.99) | 0.002 |
| Brainstem CCM | 1.05(0.20-5.62) | 0.952 |
| Presenting with hemorrhage | 4.08(0.89-18.60) | 0.070 |
| Need for feeding tube | 1.15(0.23-5.76) | 0.868 |
| Final Model |  |  |
| GCS ≤ 13 | 5.26(1.33-20.79) | 0.018 |
| mRS score ≥ 4 | 15.19(3.08-74.86) | 0.001 |
| Presenting with hemorrhage | 4.25 (1.01-17.89) | 0.049 |
| *All variables having *P*<0.05 from univariate analysis were included in multivariate analysis. The cut-off points of predictors were calculated on the basis of ROC curve analysis. †Backward stepwise regression methods were performed to create the final model whereby the least nonsignificant variable was removed from the model one at a time, until all remaining variables had *P*<0.05. | | |

| Supplementary Table 3. Multivariate analyses of postoperative intracranial infection-related factors* | | |
| --- | --- | --- |
| Components of models | Multivariate analysis† | |
|  | OR (95% CI) | *P* value |
| Model I |  |  |
| WBC ≥ 8.0×10^9^/L | 2.73(0.15-51.58) | 0.502 |
| NEU ≥ 5.2×10^9^/L | 9.10(0.49-169.65) | 0.139 |
| Final Model |  |  |
| NEU ≥ 5.2×10^9^/L | 19.39(2.19-171.71) | 0.008 |
| *All variables having *P*<0.05 from univariate analysis were included in multivariate analysis. The cut-off points of predictors were calculated on the basis of ROC curve analysis. †Backward stepwise regression methods were performed to create the final model whereby the least nonsignificant variable was removed from the model one at a time, until all remaining variables had *P*<0.05. | | |
